# Organophosphate Pesticide Exposures in Early and Late Pregnancy Influence Different Aspects of Infant Developmental Performance

**DOI:** 10.3390/toxics9050099

**Published:** 2021-04-30

**Authors:** Boonsita Suwannakul, Ratana Sapbamrer, Natrujee Wiwattanadittakul, Surat Hongsibsong

**Affiliations:** 1 Department of Community Medicine, Faculty of Medicine, Chiang Mai University, 110 Inthavaroros, Sri Phum Subdistrict, Muang District, Chiang Mai 50200, Thailand; boonsitasuwannakul@gmail.com; 2 Environmental and Occupational Health Sciences and Non Communicable Diseases Center of Excellence, Chiang Mai University, 110 Inthavaroros Road, Sriphum Subdistrict, Muang District, Chiang Mai 50200, Thailand; s_hongsibsong@hotmail.com; 3 Department of Pediatrics, Faculty of Medicine, Chiang Mai University, 110 Inthavaroros, Sri Phum Subdistrict, Muang District, Chiang Mai 50200, Thailand; natrujee.w@gmail.com; 4 School of Health Science Research, Research Institute for Health Sciences, Chiang Mai University, 110 Inthavaroros, Sri Phum Subdistrict, Muang District, Chiang Mai 50200, Thailand

**Keywords:** organophosphates, prenatal exposure, developmental performance, infants, pesticides

## Abstract

Organophosphate (OP) pesticides can transfer from mother to fetus via the placenta and amniotic fluid and may affect the development of infants. This study aims to evaluate the associations between maternal OP concentrations collected in the 1st–2nd trimester and the 3rd trimester of pregnancy and the infant developmental performance. The Screening Test of the Bayley Scales of Infants and Toddler Development, Third Edition (BSID–III screening test) was used to assess development performance at 2 and 6 months of age. Multiple regression analysis showed a negative correlation between cognitive performance at 2 months and maternal diethylthiophosphate (DETP) levels in the 1st–2nd trimester (*β* ± SE = −0.012 ± 0.004, *p* < 0.05). We also found that expressive communication and fine motor performance at 6 months were negatively associated with maternal diethyldithiophosphate (DEDTP) levels in the 3rd trimester (*β* ± SE = −0.047 ± 0.016, *p* < 0.05, and *β* ± SE = −0.044 ± 0.017, *p* < 0.05, respectively). These results suggest that maternal ethylated OP concentrations at different timing of exposure during pregnancy may influence different aspects of infant developmental performance.

## 1. Introduction

Organophosphate (OP) pesticides are a class of insecticide commonly used for agricultural purposes to control pests. Chronic low-dose exposure to OP pesticides such as occupational exposure could induce long-term adverse health effects through a non-cholinergic process. Some of these effects could include cytotoxicity, cytoarchitectural abnormalities, neuroinflammation, abnormal energy homeostasis and neurotransmission, and blood–brain barrier impairment [1]. Prenatal exposure to OP pesticides is potentially deleterious to the fetus since these chemicals can be passed to the fetus via the placenta and amniotic fluid [2]. Fetuses and young children are more susceptible to the neurotoxic effects of OP than adults as the human central nervous system, especially the brain, is undergoing rapid growth and development during the fetal period [3]. Fetuses and young children also have lower levels of detoxifying enzymes making the deactivation of OP less effective [4]. It has been shown that accumulation of OP in the placenta may disrupt fetal development. A recent study reported that maternal urinary OP concentrations collected during early and mid–pregnancy were inversely associated with fetal length and weight in mid–pregnancy [5]. Prenatal OP exposure had also been shown to have impact on birth outcomes and child neurodevelopment. Several previous studies described the relationships between maternal urinary OP metabolites and adverse birth outcomes including shortened gestation age, low birth weight, and smaller head circumference [6,7,8,9].

Repeated low-level exposure to OP pesticides during the fetal period induced long term consequences for the lifetime neurodevelopment of the offspring. These consequences included abnormal primitive reflexes in newborns and mental and motor delays among preschoolers [10,11,12,13]. However, the findings of these prior epidemiologic studies are contradictory, perhaps due to the timing of exposures. The majority of previous studies had examined the association between child neurodevelopment with only one OP exposure biomarker sample detected from maternal spot urine collected in the second or third trimester of pregnancy [14,15] or from umbilical cord blood, collected at delivery [13,16]. For example, a study by Engel et al. (2011) found that maternal total dialkylphosphate (DAP) concentrations in mid–pregnancy were negatively associated with cognitive development [14] while another study found that higher OP metabolite concentrations in a mother in late pregnancy were significantly associated with reduced motor and cognitive composite scores [15]. Some prior studies also indicated the average OP concentration from two maternal spot urine samples collected in the same or different trimesters of pregnancy were related to infant developmental performance [17,18]. A study by Eskenazi et al. (2007) found a significant negative association between the average prenatal total DAP levels collected in the 2nd trimester and Mental Development Index (MDI) scores at 24 months of age [17]. These results were inconsistent with those reported in a study by Donauer et al. (2016) which found no significant associations between average prenatal OP metabolites collected in the 2nd and 3rd trimester and cognition at 1–5 years of age [18]. One study used two maternal spot urine samples collected at the different time points of pregnancy [19]. However, these authors found no significant relationship between prenatal urinary metabolite level of OP insecticides in both 2nd and 3rd trimesters and child neurodevelopment [19].

Despite the inconsistent findings of the previous studies there is sufficient evidence to indicate that the timing of exposure to OP played a critical role in overall fetal development and neurodevelopment of infants and young children. We hypothesized that exposure to OP pesticides at different stages of pregnancy may exert different impacts on infant developmental performance. The purpose of this study was to examine the association between prenatal exposure to OP pesticides at two distinct time points and infant developmental performance among pregnant women living in an agricultural community in Chiang Mai Province, Northern Thailand. Measurements included six DAP metabolite concentrations collected once in the 1st–2nd trimester and again in the 3rd trimester of pregnancy, with infants developmental performance being analysed at 2 and 6 months of age.

## 2. Materials and Methods

### 2.1. Study Population

This birth cohort study was conducted from September 2019 to December 2020. Ninety pregnant women aged 18–35 years old and their infants were recruited. All participants lived in agricultural areas which included Galyani Vadhana, Samoeng, and Chiang Dao Districts, Chiang Mai Province, Northern Thailand. Inclusion criteria were gestation less than 28 weeks, healthy and had no major adverse health conditions which would affect child outcomes or neurodevelopment (such as thalassemia, diabetes, and hypertension), and spoke Thai as their primary language. After selection, the objectives and protocols of the study were explained to the pregnant women. All of the pregnant women had volunteered to participate and provided written informed consent before participation in the study.

### 2.2. Data Collection

The pregnant women were interviewed twice. The questionnaires were administered in Thai. In the first interview (at the first visit), following information was collected: (1) demographic data of pregnant women (age, gestation age, weight, height, education level, and family income); (2) health condition (underlying disease, medication, drugs/ alcohol use during pregnancy, utero smoke exposure); (3) source of pesticide exposure (household insecticide use, fresh fruit/ vegetable consumption, and distance from residence to farm); and (4) agricultural work characteristics (agricultural work during pregnancy and frequency of agricultural work during pregnancy). At the second interview (at 2 months after delivery), data was collected regarding the birth history (gestation age at birth, delivery procedure, complications, and serious medical condition). Birth outcome data including body weight, body length, and head circumference were extracted from medical records.

Pregnant women collected 50 mL of two spot urine samples, one at the first visit (the 1st–2nd trimester of pregnancy or gestation age < 28 weeks) and another at the second visit (the 3rd trimester of pregnancy or gestation age > 28 weeks). The urine samples were collected in polypropylene containers and stored at 2–3 °C in cooler boxes until shipment to the laboratory of the Research Institute for Health Sciences, Chiang Mai University on the day of collection. Urine specimens were aliquoted into 12 mL portions and stored at −20 °C until analysis.

### 2.3. Measurement of Urinary OP Metabolites

The gas chromatography–flame photometric detection (GC–FPD) method was used for analyzing the six nonspecific dialkylphosphate (DAP) metabolites of OP pesticides [20]. The six DAP metabolites were diethylphosphate (DEP), diethylthiophosphate (DETP), diethyldithiophosphate (DEDTP), dimethylphosphate (DMP), dimethylthiophosphate (DMTP), and dimethyldithiophosphate (DMDTP). The sum of all metabolites represented total DAP. The concentrations of all metabolites were adjusted against creatinine levels and presented in nanomoles per gram of creatinine (nmole/g creatinine). The levels of creatinine in urine samples were determined based on the Jaffe reaction [21]. The limitation of detection (LOD) ranged from 0.1 µg/L for DETP to 2.5 µg/L for DMP and the limitation of quantification (LOQ) ranged from 0.5 µg/L for DETP to 12.5 µg/L for DMP. The metabolite levels below LOD were reported as a value of LOD divided by the square root of two [22].

### 2.4. Developmental Performance Assessment

The Screening Test of the Bayley Scales of Infants and Toddler Development, Third Edition (BSID–III screening test) was used to assess infant’s developmental performance at 2 and 6 months of age. Testing was carried out by a physical therapist who had been trained in administration and scoring by an experienced pediatric physical therapist. The BSID–III screening test items included subtests of the cognition, language, and motor items of the Bayley Scales of Infants and Toddler Development, Third Edition (BSID–III). This screening test consists of five subtests: (1) cognitive subtests (33 items) that assess attention, novelty preference and habituation, problem–solving, exploration and manipulation, play, object relatedness, and concept formation; (2) receptive communication subtests (24 items) that assess auditory acuity, vocabulary development, and vocabulary related to morphological development; (3) expressive communication subtests (24 items) that assess preverbal communication, vocabulary development, and morpho–syntactic development; (4) fine motor subtests (27 items) that assess prehension, perceptual–motor integration, and functional hand skills; and (5) gross motor subtests (28 items) that assess static positioning, dynamic movement, balance, and motor planning. The number of test–items depends on the child’s age. The child’s score on the BSID–III screening test enables the practitioner to identify whether or not the child is at risk of developmental delay and further evaluation is needed. The summary score for each subtest was determined using the BSID–III subtest cut scores regarding the child’s age and classified into ‘competent’, ‘emerging’, and ‘at risk’ categories. If the child scores in the ‘competent’ category, the child is considered to be at low risk of developmental delay and in most cases does not need further evaluation. If the child scores in the ‘emerging’ category, it means that the child is considered to be at some risk of developmental delay; however, the need for further evaluation should be made in light of all information collected about the child. If the child scores in the ‘at risk’ category, it is most likely that the child needs further evaluation using an appropriate comprehensive evaluation tool such as the full BSID–III [23,24].

### 2.5. Statistical Analysis

All analyses were conducted using SPSS Statistics version 22.0. Descriptive statistics have been used to present the demographic data of pregnant women and their infants, and BSID–III screening test scores of infants at 2 and 6 months of age. Paired sample *t*-tests (or Wilcoxon test for the non–parametric test) were used to compare the differences between the urinary OP metabolites in the 1st–2nd trimester and the 3rd trimester of pregnancy. The BSID–III screening test scores were examined for the normality distribution by using histograms and we found that each subtest scores had a normal distribution. The potential demographic covariates of BSID–III screening test scores that were examined for association, and included in the multiple regression analysis if *p* < 0.2, were: maternal age, gestation age, body mass index, parity, maternal education, occupation, family income, alcohol use during pregnancy, utero smoke exposure, household insecticide use, fresh fruit/vegetable consumption, distance from residence to farm, agricultural work during pregnancy, frequency of agricultural work during pregnancy, and infant gender. A multiple linear regression model was then built to examine the association between maternal urinary OP metabolites (DEP, DETP, DEDTP, DMP, DMTP, DMDTP, and total DAP) in the 1st–2nd trimester and the 3rd trimester of pregnancy and infant developmental screening scores of BSID–III screening test (cognitive, receptive communication, expressive communication, fine motor, and gross motor scores) at 2 and 6 months of age, after controlling for the identified potential demographic covariates including maternal age, parity, maternal education, utero smoke exposure, and fresh fruit/vegetable consumption. The OP metabolites were investigated individually in each multiple linear regression model. The Benjamini–Hochberg procedure was applied for adjusting the false discovery rate (FDR) [25]. Multiple testing raw *p*-values and FDR adjusted *p*-values were reported for each association. Findings were considered statistically significant if the *p*-value was <0.05.

### 2.6. Ethical Approval

The study was reviewed and approved by the Human Ethical Committee at the Faculty of Medicine, Chiang Mai University (protocol code 221/2562 and date of approval 26 July 2019), before data collection began.

## 3. Results

### 3.1. Demographic Characteristics of the Mothers and Infants in the Agricultural Community

We included 90 healthy pregnant women in the study. Two participants were excluded from analysis due to threatened miscarriage before the second visit (in the 3rd trimester of pregnancy for collected the second spot urine sample) therefore 88 infants underwent developmental performance testing. The demographics data are reported in Table 1. Almost all participants (80.7%) were agricultural workers and most reported working in the fields during pregnancy (70.5%). 70.5% worked during the 1st trimester, 60.23% worked during the 2nd trimester, and 30.68% worked during the 3rd trimester of pregnancy. Most participants (73.9%) lived near the farmland.

The results indicated that the mean values of all birth outcomes were within the normal range of national growth reference values for Thai children [26]. However, we found that 9.1% of newborns were premature with a gestation age at birth of less than 37 weeks, 4.6% of newborns had low birth weight (<2500 g), 8.0% of newborns had low birth height (<48 cm), and 18.2% of newborns had a head circumference less than the reference range (<32 cm).

### 3.2. Urinary OP Metabolites Levels in Pregnant Women in the Agricultural Community

All urinary OP metabolite concentrations were examined for the normality distribution by using histograms and we found that each metabolite concentrations had not normally distributed. A paired Wilcoxon rank sum test was used to compare urinary OP metabolite concentrations across the two time points. We found that the number of samples with detectable levels of DMDTP was 0%. Therefore, we removed DMDTP from the analysis. The results indicated that all of the urinary OP metabolite levels in the 3rd trimester of pregnancy were significantly higher than the urinary OP metabolite levels in the 1st–2nd trimester of pregnancy (Table 2). The correlation coefficients for the different OP metabolites were evaluated by using Pearson correlation test. In the 1st–2nd trimester, the correlations between DEP and DMP (*r* = 0.633, *p* = 0.000), DETP and DEDTP (*r* = 0.352, *p* = 0.001), DEDTP and DMTP (*r* = 0.491, *p* = 0.000), and DMP and DMTP (*r* = 0.317, *p* = 0.003) were found. We also found the correlations between DEP and DETP (*r* = 0.663, *p* = 0.000), DEP and DEDTP (*r* = 0.317, *p* = 0.003), DETP and DEDTP (*r* = 0.305, *p* = 0.004), and DMP and DMTP (*r* = 0.335, *p* = 0.001) in the 3rd trimester (Table 3).

### 3.3. BSID–III Screening Test Scores of Infants at 2 and 6 Months of Age

The BSID–III screening test scores of the 88 infants at 2 and 6 months of age are shown in Table 4. When determining the infant’s performance summary score of each subtest at 2 months of age using the BSID–III subtest cut scores, we found that 27.3% of them were classified as ‘competent’ in the cognitive subtest, 67% in the receptive communication subtest, 44.3% in the expressive communication subtest, 13.6% in a fine motor subtest, and 43.2% in a gross motor subtest, while 72.7%, 33%, 55.7%, 86.4%, and 56.8% of infants respectively were categorized as at ‘emerging’ risk in the above subtest sequences. At 6 months of age, 33% of them were classified as ‘competent’ in a cognitive subtest, 60.2% in receptive communication subtest, 46.6% in expressive communication subtest, 19.3% in a fine motor subtest, and 51.1% in a gross motor subtest, while 53.4%, 35.2%, 43.2%, 70.5%, and 30.7% of infants respectively were categorized as at ‘emerging’ risk in the above subtest sequences. Lastly, 5–20% of 6 months old infants were determined to be ‘at risk’ to developmental delay in cognitive subtest (13.6%), receptive communication subtest (4.5%), expressive communication subtest (10.2%), fine motor subtest (10.2%), and gross motor subtest (18.2%).

### 3.4. Association between Maternal Urinary OP Metabolites in the 1st–2nd Trimester and the 3rd Trimester of Pregnancy and Infant Developmental Screening Scores at 2 and 6 Months of Age

Multiple linear regression analysis of the association between each maternal urinary OP metabolites (DEP, DETP, DEDTP, DMP, DMTP, and total DAP) and each infant developmental screening scores of BSID–III screening test (cognitive, receptive communication, expressive communication, fine motor, and gross motor scores) were performed with adjustment for the identified potential covariates. The potential covariates included maternal age, parity, maternal education, utero smoke exposure, and fresh fruit/vegetable consumption. The results indicated that cognitive performance at 2 months was negatively associated with maternal DETP levels in the 1st–2nd trimester of pregnancy (*β* ± SE = −0.012 ± 0.004, *p* < 0.05) (Table 5). We also found that expressive communication and fine motor performance at 6 months were negatively associated with maternal DEDTP levels in the 3rd trimester (*β* ± SE = −0.047 ± 0.016, *p* < 0.05, and *β* ± SE = −0.044 ± 0.017, *p* < 0.05, respectively) (Table 6). However, we found no significant relationships between methylated OP metabolites (DMP and DMTP) and total DAP concentrations and infant developmental screening scores of BSID–III screening test at 2 and 6 months of age.

## 4. Discussion

In this study, we reported the levels of urinary OP metabolites in the 1st–2nd trimester and the 3rd trimester of pregnancy among 88 pregnant women living in the agricultural community. We also identified the associations between prenatal OP metabolite levels in the 1st–2nd trimester and the 3rd trimester of pregnancy and infant developmental screening scores of BSID–III screening test at 2 and 6 months of age. We found that maternal DEP is the most common metabolite detected in the urine at both periods of detection. The ethylated OP metabolites have parent compounds such as chlorpyrifos, parathion, diazinon, phorate, and ethion. In Thailand, OP is the most frequently imported insecticide and is widely used in farm tasks [27]. In addition, chlorpyrifos was the most commonly found and was also the one detected at the highest level in vegetables in Thailand [28]. In the present study, most of the pregnant women reported that they had carried out agricultural work during pregnancy and lived near farmland. Therefore, the high level of urinary DEP detectable in these pregnant women might be caused by exposure to OP pesticides from the environment, fresh fruit or vegetable consumption, and/ or farm task behaviors. Also, our results indicated that maternal urinary total DAP metabolite levels in the 3rd trimester were significantly higher than in the 1st–2nd trimester (120.43 and 61.52 nmole/g creatinine, respectively). These results were inconsistent with a previous study which reported lower urinary total DAP concentration in the 3rd trimester than in the 1st–2nd trimester (14.7 and 21.5 µmol/mol creatinine, respectively) among fifty–nine pregnant farmworkers [6]. Our results had revealed a higher percentage (30.68%) women carrying out of agricultural work during the 3rd trimester than the previous study (17.3%) [6] which might result in higher exposure to OP pesticides among the participants in our study, hence the higher levels.

The results of the infant neurodevelopment assessment using the BSID–III screening test indicated that more than half of 2–month–old infants were classified as ‘emerging’ in cognitive, expressive communication, fine motor, and gross motor subtest. In addition, we found that about 5–20% of 6–month–old infants were determined to be ‘at risk’ to developmental delay in cognitive, receptive communication, expressive communication, fine motor, and gross motor domains. The proportion of developmental delay found in this study was higher than the data reported by Angsupisal et al. (2018) who assessed development using the BSID–III screening test in 64 children aged below 42 months old in an urban community of central Thailand [29]. They found only about 3–7% of the children were categorized as ‘at risk’ of developmental delays, while 70–95% of these children were generally at the lowest risk for developmental delays. It is widely recognized that OP can transfer from mother to child through the placenta and breast milk, resulting in adverse effects on the growth and development of children [2,30]. We therefore postulated that the infants in our study whose mothers lived in an agricultural community were more vulnerable to OP toxicity than the children in the urban community, resulting in a higher proportion of developmental delays.

Exposure to OP pesticides at different critical windows may have different impacts on overall fetal development including neurodevelopment in early childhood. In the present study, we found the significant associations between maternal ethylated OP concentrations at different timing of exposure during pregnancy and infant developmental performance. However, we found no significant relationships between DMP, DMTP, and total DAP and infant developmental screening scores of BSID–III screening test at 2 and 6 months of age. Our results indicated a significant negative association between prenatal DETP levels in the 1st–2nd trimester and cognitive performance at 2 months of age (*p* < 0.05). Our results were consistent with a previous study which discovered a significant negative association between the prenatal total DAP collected at the end of the 2nd trimester (26–28 weeks gestation) and cognitive development assessed with BSID–II at 12 months of age [14]. A study by Eskenazi et al. (2007) also found a significant negative association between the average prenatal total DAP levels collected in the 2nd trimester (at 14 and 27 weeks gestation) and MDI scores (cognitive composite) assessed with BSID–II at 24 months of age [17]. However, they found no significant relationships between prenatal total DAP levels and scores on MDI or PDI (motor composite) at 6 and 12 months of age. Prenatal exposure to OP insecticides in early to mid–pregnancy is potentially likely to affect the cognitive performance in the early months of life through to early childhood. However, a previous study found no significant associations between prenatal OP exposed in mid pregnancy and cognition at 1–5 years of age [18]. These authors assumed that the exposure in their cohort was too low to detect an association with the outcomes. The median of total DAP concentrations in the null study referenced here were lower than concentrations in the current study (23.7 and 54.9 nmol/g creatinine, respectively). They concluded that the level of exposure to the parent compound was not a significant risk. OP inhibit acetylcholiesterase enzyme (AChE) at nerve ending. Loss of AChE function allows accumulation of acetylcholine at synaptic cleft in brain and muscular junction, resulting in an overstimulation on muscarinic and nicotinic receptor of central, peripheral and autonomic nervous system [31,32]. To date, there is little evidence to clarify the mechanisms involved following prenatal OP exposure with regard to neurotoxic effects on the developing human brain. The human brain and nervous system begin to develop at about three weeks gestation with the closing of the neuron tube. At four weeks, the brain divided into four regions including the forebrain, midbrain, hindbrain, and optic vesicles. The rapid growth of the brain during the second trimester is followed by neuronal migration, differentiation, proliferation, and pruning throughout early childhood [33,34]. Prenatal OP exposure in these critical periods may affect the process of brain development. A previous study had investigated associations between exposure to the OP insecticide chlorpyrifos and brain morphology using magnetic resonance imaging in 40 children, aged 6–11 years and found that higher–exposure to chlorpyrifos induced brain enlargement and reduced cortical thickness in frontal, parietal, and temporal regions, resulted in cognitive functional impairment [7]. These authors suggested that prenatal OP exposure induced permanent abnormal brain morphology and function. [7]. Also, a study by Sagiv et al. (2019) indicated a negative association between total OP pesticide use within 1 km of maternal residence during pregnancy and the brain activation in the prefrontal cortex during a cognitive flexibility task of 95 adolescents [35]. The results of these studies illustrate that prenatal OP exposure affects the brain in both morphological and functional ways. However, these authors did not specify the exact critical windows of OP exposure during the pregnancy.

Our results also indicated a significant negative association between prenatal DEDTP levels in the 3rd trimester and expressive communication and fine motor performance at 6 months of age (*p* < 0.05). These results were consistent with the findings reported in a study by Kongtip et al. (2017) which found that higher total DEP and total DAP levels from mothers in the 3rd trimester of pregnancy were significantly associated with reduced motor composite scores and they also found a negative association between total DEP and cognitive composite scores of BSID–III at 5 months of age [15]. A study by Silver et al. (2017) also found that prenatal presence of naled and chlorpyrifos detected from umbilical cord blood at delivery were associated with a decrease in 9–month fine and gross motor function evaluated via the Peabody Development Motor Scale 2nd edition (PDMS−2) [13]. These results were consistent with the findings reported in a study by Rauh et al. (2006) which found a negative association between prenatal chlorpyrifos levels in umbilical cord plasma and Bayley’s psychomotor and mental development index at 3 years of age [16]. However, our results are in contrast with those reported in a study by Fluegge et al. (2016) which found no significant relationship between prenatal urinary metabolite level of OP insecticides in both 2nd and 3rd trimesters of pregnancy and child neurodevelopment assessed by BSID–II at 3 months after birth [19]. These authors surmised that the impact only in a few children who experienced clinically significant development delays might limit the clinical significance of the results. In the 3rd trimester of pregnancy, rapid myelination begins. The oligodendrocytes wrap the axons of neurons with myelin. If this process is interrupted, the myelin sheaths might not develop normally, resulting in ineffective communication between the neurons [36,37]. The child could have problems in focusing and learning abilities such as motor skills learning. Exposure to OP pesticides in late pregnancy potentially disrupted this brain development process and fetal development in this period. However, there are insufficient available published data to make a firm conclusion with regard to these associations.

The main strength of our study is that we assessed maternal OP levels in two urine samples collected from different trimesters. This enabled us to investigate time–specific associations with infant development performance and evaluate critical windows of susceptibility during the pregnancy. However, this study also has some limitations. First, the sample size was small. Our small sample size limited the statistical power of the study. We could not rule out the possibility that some of the findings may be due to chance. Second, maternal OP levels in the 1st and 2nd were considered together due to subject recruitment limitation. In the 1st trimester of pregnancy, most pregnant women in our agricultural study site did not notice that they got pregnant and had no initiate antenatal care in the hospitals. In addition, many pregnant women had a spontaneous miscarriage in this period. Therefore, we needed to recruit the pregnant women during the 1st–2nd trimester who initiated antenatal care in the hospitals to this study for the first urine sample collection. Third, DAP metabolites have a short halt–life (24–48 h.), so the urinary concentrations reflect only recent exposure [38,39]. Finally, we evaluated the infant developmental performance using the BSID–III screening test, which could identify only ‘risk’ or ‘no risk’ in developmental delay. Thus, the child who scored in the ‘at risk’ category needs further evaluation using an appropriate comprehensive evaluation tool such as the full BSID–III for specific diagnosis. All limitations should be considered to improve a future study.

## 5. Conclusions

The data from this study suggest that maternal ethylated OP concentrations at different timing of exposure during pregnancy may influence different aspects of infant developmental performance. Prenatal OP exposure in the 1st–2nd trimester of pregnancy was inversely associated with cognitive performance at both 2 and 6 months of age. In addition, prenatal OP exposure in the 3rd trimester of pregnancy was inversely associated with communication and motor performance at 6 months of age. However, the underlying mechanisms behind these correlations are not fully understood and future research needs to focus more on the damaging processes behind these findings. Intervention and prevention strategies regarding prenatal pesticide exposure in the agricultural community are necessary to prevent long-term adverse health effects in children.

## Figures and Tables

**Table 1:** Demographic data of the mothers and infants in the agricultural community (*n* = 88).

Parameters		*n* (%) or Mean ± SD
**Maternal characteristics**		
Age (years), mean ± SD		24.32 ± 4.90
Gestation age (weeks), mean ± SD	1st–2nd trimester 3rd trimester	19.33 ± 6.65 31.26 ± 2.11
Body mass index (kg/m^2^), *n* (%)	<18.5 18.5–24.9 25–29.9 ≥30	3 (3.4) 51 (58.0) 26 (29.5) 8 (9.1)
Parity, *n* (%)	1st child 2nd child 3rd child	26 (29.5) 40 (45.5) 22 (25.0)
Education, *n* (%)	Junior high school or less Senior high school or higher	57 (64.8) 31 (35.2)
Occupation, *n* (%)	Agricultural workers Other	71 (80.7) 17 (19.3)
Family income (Baht/month), *n* (%)	<4500 4500–9000 >9000	44 (50.0) 35 (39.8) 9 (10.2)
Alcohol use during pregnancy, *n* (%)		1 (1.1)
Drugs use during pregnancy, *n* (%)		0 (0)
Utero smoke exposure, *n* (%)	Yes No	38 (43.2) 50 (56.8)
Household insecticide use, *n* (%)		46 (43.4)
Fresh fruit/ vegetable consumption (days/week), *n* (%)	7 4–6 1–3	50 (47.2) 12 (11.3) 26 (24.5)
Distance from residence to farm (km), *n* (%)	0–5 >5	65 (73.9) 23 (26.1)
Agricultural work during pregnancy, *n* (%)	Yes No	62 (70.5) 26 (29.5)
Frequency of agricultural work during pregnancy (days/week), *n* (%)	5–7 4 or less	53 (60.2) 35 (39.8)
**Infant characteristics**		
Delivery procedure, *n* (%)	Normal labor Cesarean section	74 (84.1) 14 (15.9)
Infant gender, *n* (%)	Male Female	35 (39.8) 53 (60.2)
Gestation duration (weeks), mean ± SD		38.28 ± 1.72
Birth weight (g), mean ± SD		3087.76 ± 435.25
Birth height (cm), mean ± SD		51.45 ± 2.96
Head circumference (cm), mean ± SD		32.72 ± 1.67

**Table 2:** Urinary OP metabolites (nmol/g creatinine) in the 1st–2nd trimester and the 3rd trimester of pregnancy in pregnant women (*n* = 88) in the agricultural community.

OP Metabolites	1st–2nd Trimester				3rd Trimester				*p*-Value
	% Samples Detectable	GM	Median	Min–Max	% Samples Detectable	GM	Median	Min–Max	
DEP	64.77	6.89	6.82	<LOD-807.14	67.05	15.04	11.82	<LOD-314.17	0.000 **
DETP	51.14	2.47	1.59	<LOD-167.33	65.91	7.52	6.20	<LOD-411.32	0.000 **
DEDTP	3.41	1.68	1.63	<LOD-19.27	11.36	3.10	2.44	<LOD-62.84	0.000 **
DMP	6.82	33.25	31.12	<LOD-1086.81	4.55	47.39	40.65	<LOD-971.38	0.007 *
DMTP	5.68	4.25	4.25	<LOD-20.45	21.59	8.23	7.34	<LOD-1358.41	0.000 **
Total DAP	70.45	61.52	54.92	<LOD-1817.12	75.00	120.43	103.52	<LOD-2040.16	0.000 **

Note: GM, geometric mean; Min, minimum; Max, maximum; LOD, limit of detection; DEP, diethylphosphate; DETP, diethylthiophosphate; DEDTP, diethyldithiophosphate; DMP, dimethylphosphate; DMTP, dimethylthiophosphate; DMDTP, dimethyldithiophosphate; * *p* < 0.01; ** *p* < 0.001. Total *DAP* = DEP ± DETP ± DEDTP ± DMP ± DMTP.

**Table 3:** Pearson correlation coefficient of DAP metabolites.

DAP Metabolites	DEP		DETP		DEDTP		DMP		DMTP	
	R	*p*-Value	r	*p*-Value	R	*p*-Value	R	*p*-Value	R	*p*-Value
**1st–2nd trimester**										
DEP	–	–	0.044	0.681	0.054	0.617	0.633	0.000 **	0.151	0.161
DETP	–	–	–	–	0.352	0.001 **	−0.031	0.773	−0.018	0.865
DEDTP	–	–	–	–	–	–	0.125	0.245	0.491	0.000 **
DMP	–	–	–	–	–	–	–	–	0.317	0.003 **
DMTP	–	–	–	–	–	–	–	–	–	–
**3rd trimester**										
DEP	–	–	0.663	0.000 **	0.317	0.003 **	−0.001	0.996	0.054	0.615
DETP	–	–	–	–	0.305	0.004 **	−0.006	0.954	0.148	0.169
DEDTP	–	–	–	–	–	–	0.058	0.590	−0.011	0.922
DMP	–	–	–	–	–	–	–	–	0.335	0.001 **
DMTP	–	–	–	–	–	–	–	–	–	–

** Correlation is significant at the 0.01 level (2–tailed).

**Table 4:** The BSID–III screening test scores of infants at 2 and 6 months of age (*n* = 88).

Subtest	2 Months of Age				6 Months of Age			
	Mean	Median	SD	Range	Mean	Median	SD	Range
Cognitive	1.90	2	1.29	0–5	5.44	6	1.88	2–10
Receptive communication	3.11	3	1.19	0–5	5.86	6	1.66	2–10
Expressive communication	1.80	1	1.29	0–6	4.14	4	1.53	0–8
Fine motor	1.72	2	0.80	0–4	5.06	5	1.53	2–8
Gross motor	1.94	2	1.37	0–4	6.35	6.5	2.18	1–11

**Table 5:** Association between maternal urinary OP metabolites in the 1st–2nd trimester and infant developmental screening scores using the BSID–III screening test at 2 and 6 months of age.

OP Metabolites	Cognitive			Receptive Communication			Expressive Communication			Fine Motor			Gross Motor		
	β ± SE	Raw *p*-Value	FDR Adjusted *p*-Value	β ± SE	Raw *p*-Value	FDR Adjusted *p*-Value	β ± SE	Raw *p*-Value	FDR Adjusted *p*-Value	β ± SE	Raw *p*-Value	FDR Adjusted *p*-Value	β ± SE	Raw *p*-Value	FDR Adjusted *p*-Value
**2 months of age**															
DEP	0.000 ± 0.002	0.968	0.063	−0.001 ± 0.001	0.546	0.063	−0.002 ± 0.002	0.187	0.063	0.001 ± 0.001	0.625	0.063	−0.000 ± 0.002	0.993	0.063
DETP	−0.012 ± 0.004	0.005	0.030 *	0.004 ± 0.004	0.350	0.063	0.006 ± 0.005	0.218	0.063	0.002 ± 0.003	0.451	0.063	−0.002 ± 0.005	0.727	0.063
DEDTP	0.040 ± 0.050	0.432	0.063	−0.004 ± 0.047	0.926	0.063	−0.01 ± 0.052	0850	0.063	−0.006 ± 0.034	0.871	0.063	−0.002 ± 0.056	0.968	0.063
DMP	0.000 ± 0.001	0.900	0.063	0.000 ± 0.001	0.965	0.063	0.00 ± 0.001	0.141	0.063	0.000 ± 0.001	0.496	0.063	0.001 ± 0.001	0.586	0.063
DMTP	−0.018 ± 0.034	0.599	0.063	0.007 ± 0.031	0.828	0.063	−0.048 ± 0.033	0.153	0.063	0.005 ± 0.022	0.082	0.063	0.061 ± 0.036	0.099	0.063
**6 months of age**															
DEP	−0.002 ± 0.002	0.285	0.063	0.003 ± 0.002	0.084	0.063	0.002 ± 0.002	0.196	0.063	0.003 ± 0.002	0.088	0.063	0.001 ± 0.003	0.660	0.063
DETP	0.000 ± 0.008	0.859	0.063	0.001 ± 0.006	0.859	0.063	−0.00 ± 0.006	0.486	0.063	−0.001 ± 0.006	0.873	0.063	−0.008 ± 0.008	0.334	0.063
DEDTP	0.070 ± 0.075	0.353	0.063	−0.013 ± 0.066	0.847	0.063	−0.00 ± 0.061	0.952	0.063	0.009 ± 0.063	0.884	0.063	0.00 ± 0.087	0.998	0.063
DMP	0.001 ± 0.001	0.352	0.063	0.002 ± 0.001	0.103	0.063	0.001 ± 0.001	0.357	0.063	0.002 ± 0.001	0.063	0.063	0.001 ± 0.001	0.674	0.063
DMTP	0.032 ± 0.049	0.519	0.063	−0.025 ± 0.043	0.569	0.063	−0.060 ± 0.039	0.128	0.063	0.016 ± 0.041	0.699	0.063	0.022 ± 0.057	0.701	0.063

Note: adjusted with maternal age, parity, education, utero smoke exposure, and fresh fruit/vegetable consumption; β beta; SE standard error; * *p* < 0.05.

**Table 6:** Association between maternal urinary OP metabolites in the 3rd trimester and infant developmental screening scores using the Bayley III screening test at 2 and 6 months of age.

OP Metabolites	Cognitive			Receptive Communication			Expressive Communication			Fine Motor			Gross Motor		
	β ± SE	Raw *p*-Value	FDR Adjusted *p*-Value	β ± SE	Raw *p*-Value	FDR Adjusted *p*-Value	β ± SE	Raw *p*-Value	FDRAdjusted *p*-Value	β ± SE	Raw *p*-Value	FDR Adjusted *p*-Value	β ± SE	Raw *p*-Value	FDR Adjusted *p*-Value
**2 months of age**															
DEP	−0.006 ± 0.002	0.053	0.063	−0.00 ± 0.002	0.576	0.063	0.000 ± 0.002	0.831	0.063	−0.001 ± 0.001	0.279	0.063	−0.001 ± 0.002	0.757	0.063
DETP	−0.003 ± 0.002	0.065	0.063	−0.001 ± 0.002	0.576	0.063	−0.001 ± 0.002	0.555	0.063	0.001 ± 0.001	0.546	0.063	−0.001 ± 0.002	0.726	0.063
DEDTP	−0.021 ± 0.018	0.097	0.063	−0.008 ± 0.013	0.513	0.063	−0.020 ± 0.014	0.136	0.063	−0.013 ± 0.009	0.173	0.063	0.006 ± 0.016	0.692	0.063
DMP	0.000 ± 0.001	0.865	0.063	−0.002 ± 0.001	0.088	0.063	0.000 ± 0.001	0.638	0.063	0.000 ± 0.001	0.892	0.063	0.000 ± 0.001	0.802	0.063
DMTP	−0.001 ± 0.001	0.278	0.063	−0.001 ± 0.001	0.346	0.063	−0.001 ± 0.001	0.333	0.063	−0.001 ± 0.001	0.404	0.063	0.001 ± 0.001	0.623	0.063
**6 months of age**															
DEP	0.000 ± 0.003	0.942	0.063	−0.001 ± 0.003	0.759	0.063	0.002 ± 0.003	0.419	0.063	0.001 ± 0.003	0.666	0.063	0.000 ± 0.004	0.861	0.063
DETP	0.002 ± 0.003	0.389	0.063	0.000 ± 0.002	0.865	0.063	−0.001 ± 0.002	0.731	0.063	0.003 ± 0.003	0.336	0.063	0.001 ± 0.003	0.691	0.063
DEDTP	−0.020 ± 0.021	0.635	0.063	−0.038 ± 0.018	0.103	0.063	−0.047 ± 0.016	0.007	0.042 *	−0.044 ± 0.017	0.008	0.04 *	−0.025 ± 0.024	0.303	0.063
DMP	0.001 ± 0.002	0.563	0.063	0.001 ± 0.001	0.344	0.063	0.000 ± 0.001	0.722	0.063	0.001 ± 0.001	0.137	0.063	0.001 ± 0.002	0.443	0.063
DMTP	0.001 ± 0.001	0.440	0.063	−0.001 ± 0.001	0.495	0.063	−0.001 ± 0.001	0.458	0.063	−0.001 ± 0.001	0.521	0.063	0.000 ± 0.002	0.864	0.063

Note: adjusted with maternal age, parity, education, utero smoke exposure, and fresh fruit/vegetable consumption; β beta; SE standard error; * *p* < 0.05.
